# Factors associated with mental health outcomes among medical residents exposed to COVID-19

**DOI:** 10.1192/bjo.2021.617

**Published:** 2021-06-18

**Authors:** Mohamed Adil Shah Khoodoruth, Sami Ouanes, Malek Smida, Zerak Al-Salihy, Saleem Al Nuaimi, Widaad Nuzhah Chut-kai Khoodoruth, Adeel Ghaffar, Mohammed Faisal Hamad Mohammed

**Affiliations:** 1 Psychiatry Hospital, Hamad Medical Corporation; 2 Hamad Medical Corporation

## Abstract

**Aims:**

The aims of our study were to assess and to examine i. the psychological impact of the COVID-19 pandemic on medical residents working on the front and second line, ii. the association between coping strategies, resilience and optimism and different mental health outcomes like stress, anxiety, and depression among the medical residents’ workers during the COVID-19 pandemic, and iii. the coping strategies used on the same sample with consideration of different factors like seniority, frontliner, gender and coping style.

**Method:**

An electronic survey was sent to all medical residents in Qatar. Depression, anxiety and stress were assessed by the Depression, Anxiety and Stress Scale – 21 Items. Professional quality of life was measured by the Professional Quality of Life measure. The coping mechanisms were assessed with the Brief-COPE, resilience by the Brief Resilience Scale, and optimism by the Revised Life Orientation Test (LOT-R).

**Result:**

Of the 640 medical residents contacted, 127 (20%) responded. A considerable proportion of residents reported symptoms of depression (42.5%), anxiety (41.7%) and stress (30.7%). Multivariate analysis of variance showed significant effects of seniority in residency, with junior residents having poorer outcomes. In addition, there was a statistically significant interaction effect with moderate effect sizes between gender and working on the front line, as well as gender, working on the front line and seniority, on mental health outcomes. The most commonly used coping strategies were acceptance, religion, and active coping. The least reported coping strategies were substance use and denial. Avoidant coping style scores were higher among junior residents (p = .032) and non-COVID-19 frontliners (p = .039). Optimism LOT-R score was higher in senior than in junior residents (p < .001). Another important finding is that optimism and resilience were associated with better mental health outcomes. In addition, we find that avoidant coping style is highly associated with depression.

**Conclusion:**

The COVID-19 pandemic may have a negative impact on junior residents’ mental health. Preventive measures to reduce stress levels and easy access to professional mental health services are crucial. This study also raises awareness among residency programs on the psychological and coping responses and strategies of medical residents.

